# Stunting Rates in a Food-Rich Country: The Argentine Pampas from the 1850s to the 1950s

**DOI:** 10.3390/ijerph17217806

**Published:** 2020-10-25

**Authors:** Ricardo D. Salvatore

**Affiliations:** Departamento de Estudios Históricos y Sociales, Universidad Torcuato Di Tella, Argentina, Figueroa Alcorta 7350, C1428BCW Ciudad de Buenos Aires, Argentina; rdsalva@utdt.edu

**Keywords:** anthropometric history, stunting rates, malnutrition, food-production, Argentina

## Abstract

Little is known about the effects of malnutrition rates in the long-run. Applying the methodology recommended by the World Health Organization, this study estimates stunting rates for Argentine adult males from the 1850s to the 1950s. We use five large samples of army recruits, prison inmates, militiamen, and electoral records totaling 84,500 cases. These samples provide information about height in Buenos Aires province and the Pampa region, the most fertile, food-producing area of the country. As the study shows, estimated stunting rates remained stable from the 1850s to the 1880s and then declined persistently until the 1950s. The total decline was substantial: if fell from 15.3% in the 1870s to 5.6% in the 1940s, then stagnated. In this 95-year period, stunting rates went from “medium” to “low” levels in the WHO classification of malnutrition intensity. At the end of our study period (the 1950s) the Pampa’s malnutrition rate was only 3.5 to 4 percentage points above contemporary estimates for well-developed economies in Europe and North America. A significant expansion in the region’s production of grains and beef (food availability), combined with a sustained decline in infant mortality (increased health) were probably the two main underlying factors of this long-tern reduction in malnutrition. Yet, this association remains to be determined.

## 1. Introduction

For the last twenty years, Anthropometric History has produced a rich set of publications about the biological wellbeing of diverse populations in the Latin American past [1,2,3]. First in the agenda was the quest for new data to generate long-term estimates of average heights [4,5,6,7,8,9,10,11,12,13,14,15]. Later interest shifted to estimating the size of social and regional inequalities in biological wellbeing [16,17,18,19]. More recently authors have focused their attention on the question of past inequalities in the access of food and health by poor children, women and ethnic/racial minorities [20,21,22]. While in Europe and North America scholars had paid attention to the question of children’s health and nutrition in the past (at least since the 18th century), in Latin America historical studies on children’s heights are still rare [23,24,25,26,27,28,29,30,31,32].

For Argentina, prior to 1990, there are a limited number of studies, conducted by pediatricians and nutritionists, based on small samples of schoolchildren. The limited geographic coverage and small sample size of these studies have prevented researchers from having a long-term perspective on the evolution of stunting rates. Starting in the 1990s there has been an increasing number of studies on child malnutrition in Argentina (a combination of clinical studies and national health surveys), all of them focused in contemporary populations and motivated by the need to devise policies to alleviate poverty and malnutrition [33,34,35,36,37,38]. As far as I know, there is no study of the long-run evolution of malnutrition in the country.

This study attempts to estimate rates of moderate and severe malnutrition from the 1850s to the 1950s, using five large historical databases collected by the author. All of these samples (stemming from army, militia, prison, and electoral records) provide evidence of the evolution and distribution of adult male heights in Argentina. Our estimates show that, after 25 years of stable malnutrition rates (1855–1880), there ensued a long and persistent reduction of malnutrition rates that extended from the 1880s to the 1950s. This sustained fall in malnutrition took place in the midst of significant transformation in Argentine society, politics and the economy; among them, the great agrarian expansion that led to a period of rapid economic growth, based on the exports of foodstuffs (1880–1920). The decline in malnutrition also extended to the first phase of industrialization (1930–1960), a period in which state policies were detrimental to agriculture. The publication of these estimates is intended to stimulate further research on the conditions and factors explaining this long-run reduction in malnutrition, and also to invite other scholars to join the effort to generate stunting rates for the past 100 or 150 years.

## 2. A Food-Rich Economy

Argentina was known as a country which had experienced a great agrarian modernization (during the period 1880–1920), as a result of which it became a wealthy food-exporting economy. Travelers, diplomats, and publicists considered Argentina a country of “meat-eaters”, linking the old story of 18th century “gauchos” who lived mostly on beef from stolen cattle with indicators of high per-capita meat consumption in the early20th century. In the period covered by this study there was a revolution in the productivity of wheat farming (that involved seed selection, wider iron-plows, mechanical threshers, and the expansion of farm size), and also a transformation of cattle-raising from old “estancias” producing very-lean creole cattle to modern cattle ranches that raised cross-breed cattle to sell steers rich in meat and fat. By the 1920s, Argentina was one of the first five global producers of wheat and other grains, while Argentine refrigerated beef, produced in modern meat plants owned by British and North-American firms, was highly regarded by European consumers. I called the Pampa region a “food-rich” economy because this prairie land generated since mid-1880 massive surpluses of food (grain and meat) intended for global markets. This relative abundance of food persisted into the postwar period, amidst unfavorable state policies of price and exchange controls, regulated imports of machinery, and declining relative prices.

Available statistics of per-capita meat and grain consumption tend to over-estimate the dietary abundance of Argentine consumers. Nonetheless, it is true that meat and bread were relatively cheap in comparison to Europe and the United States. Based on official statistics of the period 1935–1940, E. Llorens claimed that Argentina was the country that consumed the highest quantity of meat in the world: 136 kg per year, well above the average annual consumption of countries such as the United States (60 kg), United Kingdom (64 kg), Denmark (51 kg) and Switzerland (47 kg). The only countries that came close to Argentina were New Zealand (107 kg) and Australia (91 kg). Something similar occurred with bread consumption: Argentine average consumption of bread was the equivalent of 152 kg of wheat per year. Only France (183 kg) and Italy (159 kg) exceeded Argentina in bread per-capita consumption, and Australia was at the same level (151 kg). The rest of European countries and the United States were within the range of 100–140 kg per year [39]. This abundance of food apparently extended to urban working-class families. According to contemporary budget studies Llorens estimated that a working-class family in Buenos Aires consumed 84 kg of bread and 57 kg of meat per-capita per annum. While the figure of Argentine meat consumption was second to Montevideo’s figure, other cities such as Medellín and Bogotá consumed more bread than the “Queen of the Plata.” Overestimation of per-capita consumption and large inequalities in income and wages across social classes and internal regions should warn us against associating the notion of a “rich-food economy” with an egalitarian, well-fed population. Though a food-rich economy, the Argentine pampas generated stunted children. We need to estimate the historical magnitude of this deficiency in biological wellbeing. 

The period chosen (1855–1959) involved important transformations in Argentina’s society, institutions, and economy. At the time of the constitutional arrangement of 1860, the country was still divided into two sovereign entities: the State of Buenos Aires and the Argentine Confederation. Their unification in 1862 reduced the frequency of internal armed conflicts, yet it was not until the early 1880s that the country could enjoy relative harmony among the provinces and security on its frontiers. During the 1860s and 1870s Indian attacks to frontier farmers and cattle-raisers were quite intense in the Pampa region. In 1880 the Capital city was federalized and with this came the centralization of government and the end of regional rebellions. A year earlier, a vast military campaign defeated the last resistance of indigenous peoples in northern Patagonia, freeing a vast territory to the south and west for the cultivation of grain and the raising of cattle and sheep (for a summary view of Argentine economic history see [40,41,42,43,44,45].)

The centralization of government authority and the control of a national territory combined with the massive arrival of European immigrants and capital to usher a period of agrarian export bonanza that historians call the Argentine “Golden Age” (1880–1920). A new immigrant work force, foreign investment, and stable political institutions generated rapid economic growth for more than 30 years. WWI stopped the inflow of European immigrants and drastically reduced the inflow of foreign credit and investment, slowing down growth. During the 1920s exports of grain achieved new heights, while the country conquered stable markets for its chilled beef and mutton. The Pampa region then became one of the “granaries” of the world. The Great Depression produced a dramatic fall in the country’s main exports, while the change in relative prices and new exchange and import controls facilitated the emergence of an industrial sector. Industrial production and employment made possible an early recovery and produced rapid growth in the second half of the 1930s. Industrialization proceeded at fast pace in the 1940s and 1950s due to new availability of credit, state protection to industry, rising real wages, and increased investments in energy and transportation. Furthermore, during 1930–1960 the country’s demographic outlook was radically transformed: masses of provincial migrants moved to Buenos Aires and other provinces of the Littoral in search for salaried employment and the amenities of city life.

Our estimates of stunting rates relate mostly to the Humid Pampas, including the provinces of Buenos Aires, Entre Ríos, Santa Fe, and Córdoba. The Pampa area benefited the most from the export bonanza of the period 1880–1914 and also from the industrialization and rapid urbanization of 1930–1960. These concurrent processes of industrialization and urbanization increased income and net-nutrition inequalities, among regions as well as among socio-economic groups. [16] The Interior provinces of Argentina lost part of its human capital and failed to participate of industrialization. At the present these provinces suffer from persistent poverty and show significantly higher rates of malnutrition than the Pampa region. The interior provinces are not considered here—mainly because the lack of height distributions for these provinces since the 1850s. 

## 3. Data Sources

We estimated malnutrition rates based on distributions of adult heights for the period 1855–1959. The estimates come from five large samples the author has collected over time. A brief description of these samples is provided next. 

### 3.1. National Guards b.1857–1883

In the Archivo Histórico de la Provincia de Buenos we located the papers of the General Inspection of Militias, which contained a book on the 1902 enrolment of the National Guards. This register included men 19 to 45 years old enrolled in 17 districts (partidos) of Buenos Aires province. They had been born in almost every district of the province, which at the 1881 census was divided into 79 partidos (separating those districts for which we had more than 40 observations in each extreme (b1857–1865 and b1875–1883), we could estimate reliable means for at least 35 of the 79 partidos.) Our sample contained information on the height of 11,158 men born between 1857 and 1883. This total represented 19.3% of all men enrolled in the militias; and 21.5% of the male native population of the province in 1881. These men were 99 percent Argentine; of them 80.2% were born in Buenos Aires province, 9.6% in the Capital city, and 9.8% in the Interior provinces. The sample represented adequately the three different zones of the province: 57.0% the Northern zone; 35.6% the Central zone; and 5.4% the Southern zone-t-he latter territory was controlled by indigenous peoples until 1879. While this database did not contain information about occupation, race, and reading skills, in other enrolment books of the same institution, we found more complete information for a sample of 9 districts, representing men born in the same period. Of them 68% were rural peons, 3.5% were craftsmen and mechanics, 6.8% employees, 3.8% merchants, 1.7% students and professionals, 8.9% farmers, and 6.2% landowners. See [46].

### 3.2. Citizen-Soldiers from the Pampa Region b.1874–1910

In the Army General Archive, we found information about 20,925 men who had previously enlisted in the army and that, due to the Electoral Law of 1926, were obligated to register in the following year. These men had been born in Buenos Aires province (94.7%), the Capital city (2.0%), and the Interior provinces (3.3%) between 1874 and 1910. We collected data for birth years 1874–1875; 1879–1880; 1884–1885; 1889–1890; 1894–1895; 1899–1900; 1904–1905; and 1909–1910 from a representative sample of 21 rural districts of Buenos Aires province. The sample produced information for individuals born in at least 50 different partidos. Their occupations were: 46.6% unskilled laborers (peons), 6.4% skilled workers (craftsmen and mechanics), 11.3% employees, 32.4% independent producers (farmers and merchants), 3.3% students, teachers and professionals, and 1.7% landowners. Like the previous sample, this one reflects the biological condition of 18 to 18.5 year-old men, most of them born and raised within the territory of Buenos Aires province. Most had basic reading skills (79% of them), while the rest (21%) were illiterate. According to the physicians who checked them, 8.4% of them had some physical incapacity or illness that made them unable for service (in the case they were called to arms). See [47].

### 3.3. Patricios Battalion Sample b.1901–1943

This is a sample of 29.430 men who presented themselves to the medical check-up and registration, within the three months following their 18th birthday, to comply with the Compulsory Military Service law of 1901. A sample of 800 to 1200 observations was selected for each year. These recruits were all born in Argentina and had enlisted in the surroundings of Buenos Aires city (they went to register to the Patricios Regiment). More than half of these recruits (54.4%) had been born in the Pampa region (most of them in Buenos Aires province); the rest in the interior provinces (36.0%) and in the Capital city (9.6%). Though most enjoyed good health, an important 19.3% was considered either “inept” or “deficient” for service. These recruits, who entered the military after 1920, had very high literacy rates (97%). Such high literacy rates reflected both the army’s soft definition of literacy (the ability to sign one’s name) and the early success of the alphabetization campaigns in the region. Their declared occupations were: 24.7% were unskilled laborers; 26.1% skilled workers (craftsmen and mechanics), 28.2% employees, 8.7% independent producers (farmers, merchants or industrialists), and 11.7% students or teachers. This sample can be considered representative of 18 year-old males in the Pampa region, with an important component coming from the interior provinces. See [5].

### 3.4. Inmates from Buenos Aires Prisons b.1885–1959

This is a large sample of prisoners extracted from the Prison Register books of five important penal institutions under the jurisdiction of Buenos Aires province. These books were found in the Archivo y Museo del Servicio Penitentiario Bonaerense at La Plata. The sample contains 12,783 observations of delinquents serving prison terms at penal institutions in Azul, Dolores, Bahía Blanca, La Plata, Mercedes, Olmos, and San Nicolas. Inmates had been born in Buenos Aires city (10.3%), in Buenos Aires province (67.9%), in the rest of the Pampa region (10.5%), and in the Interior provinces (11.2%), Besides heights, occupation, and place of birth, these records contain information about race, parents’ origins and schooling of inmates. Inmates over-represent the working-class population. Unskilled laborers, skilled workers, and employees constituted 89.5% of the prison population. On the other hand, the middling sectors were under-represented (industrialists, merchants, farmers and students added up 8.7% of the sample), while the upper class was almost inexistent (0.5%). Most of the prisoners were white (50.7%) or trigueños (40.6%) (a “trigueño” is a sun-tanned mestizo, whiter than a “moreno” (black) but darker than a European White. The name refers to his skin being like the color of a wheat spike.) Like army recruits, many prisoners said they were literate (86.7%), yet only 13% have attended school enough years to complete elementary education and only 3% had started secondary school. See [48]. Compared with army recruits, prison inmates were older, less educated, and taller.

### 3.5. Recruits from the Pampa Region b.1916–1951

This is part of a larger sample intended to measure the wellbeing of Argentines in the inter-war period. It is composed of 10,112 recruits born in the Pampa region between 1916 and 1951. 18-year old recruits were literate; 95% of them said they knew how to write. During the interwar period many small cities had emerged in the Pampa region, Hence, most of these recruits were urban rather than rural: 61.2% of recruits lived in cities between 5000 and 25,000 inhabitants, and another 15.1% lived in cities between 25,000 and 100,000 inhabitants. Workers represented 67.2% of the sample. During this period, the proportion of unskilled laborers not so overwhelming in comparison to skilled workers and employees as it had been before 1914. The middling sector (independent producers and students and teachers) was important, representing 32.7% of the sample. In health, these recruits were not different from the recruits of the Patricios Battalion; for 20.4% of them were considered “inept” or “deficient” for service. See [6,16].

These five samples, taken together add up to more than 84,400 records of heights. To the best of my knowledge, they can be taken as representative of the biological condition of Buenos Aires province (both young men of ages 18–19 and older men of ages 20 to 45) and, to a lesser extent, they represent the rest of the Pampa region. For this region, we have enough information to cover 95 years of Argentine anthropometric history. Not for the rest of the country, where our information is less systematic and complete (for a study of heights in the Argentine Northwest, see [17]). Here are the basic statistics for the five height samples. See Table 1.

Except for the Prisoner’s sample, the rest of samples do not present any considerable heaping and its observations are distributed in quasi-normal or Gaussian curves. In all cases, the data refers to males born in Argentina, not to immigrants. During the period 1880–1920 Argentina received massive inflow of migrants from Europe, most of them Italians and Spaniards who were shorter in average than the native-born. I do not claim this data to be representative of all the male population of the Pampa region; only of native Argentines. It makes little sense to include the immigrant population in the sample, to the extent that they were born (and spent their infancy) in their countries of origin—that is, their heights say nothing about living conditions in the Argentine Pampas.

The five samples I used are representative of the native-born population of the Pampa region, in particular of Buenos Aires province. Three of our samples, involving army recruits and citizens, due to their large size, include individuals from all socio-economic groups, though not in fixed proportions. Yet instead of reducing the sample size by considering separate distributions for workers, middle-sectors, and upper-class individuals, I preferred to work with the widest possible distributions. The samples involving army recruits do not suffer from truncation, for there was no minimum height-requirement for the compulsory military service. Every 18-year old male, within the three months following his 18th birthday had to show up at the nearest army unit and have his medical check-up (perhaps, a very small percentage of this age-specific growth could not travel to an army unit, if they were really “out there” in the confines of the nation: high mountains, difficult to reach forests, etc. This was not the case in the Pampa region where most districts had a nearby army-unit). Later on, a national lottery determined who would spend time in military instruction and who would not. The same could be said of National Guard militiamen: every native-born of ages 19 to 49 had to go to the place of enlistment, usually in a nearby city within their own “partido.” Enlisting was an obligation, but this did not imply going into combat; only a small part of those enrolled ever saw any military action. Later on, the 1927 Electoral Registry made into law a precept that was implicit from the late 19th century and became explicit in the 1912 electoral reform: only those who had made the military service were entitled to vote (and were listed in the electoral roster). For that reason, I do not think that our samples have the common selectivity problems some scholars attribute to military databases: truncation; different schooling; and social class [49,50].

## 4. Methodology

There are two key anthropometric measures of malnutrition: heights for age; and weight for age. Low height for age indicates “stunting”—a retardation of normal growth—while low weight for age is an indicator of “wasting”; a temporary, yet acute condition [45,46,51]. Both are associated with insufficient nutrition, infectious diseases, environmental insults, and excessive labor exertion. Yet it is mostly stunting the condition that, stemming from an insult to health and nutrition during infancy, can become permanent and have an impact in mid-life health, increasing the risk of chronic diseases and mortality. Since the 1960s physicians, nutritionists and epidemiologists began to speak of a condition called P.E.M., or protein-energy malnutrition. Both wasting and stunting are implicated in this condition. Sources of historical data on heights are more abundant than data on weight. For Argentina in particular, pre-1960 data on weights is very rare and data on children heights is spotty and selective- So we are forced to estimate stunting using adult heights. Though originally devised to measure malnutrition for children under five, we believed it is possible and reasonable to apply to adult heights the anthropometric instruments devised to examine malnutrition in children. The distribution of adult heights, we suggest, can be thought of as a mere transformation of the distribution of children heights (for a discussion on the importance of the tempo of the net-nutrition insult on the pattern of adolescent growth, see [52]).

In 1986 a Growth and Development Committee at the Argentine Pediatric Society (S.A.P.) asked fellow pediatricians to work towards the construction of national standards of child growth. Members of the committee thought that the existing international standards were unachievable for Argentine children; consequently, they began promoting the calculation of more realistic standards. The new tables should reflect children’s normal pattern of growth under the best of local conditions. The first study [53], based on samples from Córdoba and La Plata was published in 1987 and was adopted later by the S.A.P. through its first Guide for Evaluating Physical Growth. A second edition of this guide, published in 2001, has been used since then by pediatric doctors across the country as a main tool to evaluate the normal or pathological development of Argentine children. [54] The standards disseminated by the S.A.P. established that a normal, well-fed and healthy 18-year old at the 50th percentile should measure 172.6 cm with a SD of 6.85 cm [54,55]. The third edition of SAP’s Guide, published in 2007, incorporated the findings of new studies, among them the article by Del Pino et.al [36], based on a sample of 1900 adolescents across the nation. Though the authors noticed a small variation in the heights of young men and women, they concluded that the standards of the 2001 edition of the Guide should remain unchanged.

Meanwhile in the North-Atlantic, in the 1980s and 1990s new standards of growth were being constructed and disseminated world-wide. First, 1977 the National Center for Health Statistics estimated modern growth standards based on U.S. samples [56]. It was thought that US-Americans, then one of the tallest populations in the world, represented the potential for human growth [57]. Since 1979 the NCHS reference for child growth has been widely used to compare the nutritional status of infants throughout the world. A few years later, the World Health Organization defined its own standards of child-growth, publishing two milestone studies of human growth in 1983 and 1995 [58,59] (when in 1996 Steckel recommended historians to use U.S. heights and weights as standards for modern growth, he was referring to the standards generated by the NCHS in 1977 [60]). Responding to the criticism raised by many scholars and physicians about the local nature of the first estimates, in 2006 WHO published new standards of growth for children under five years based on studies conducted in Brazil, Ghana, India, Norway, Oman and the USA between 1997 and 2003 [61,62]. These standards were complemented a year later with tables containing normal or expected heights and weights for adolescents [63,64]. This revised WHO standards established the mean height for the percentile 50th of an 18-year old male at 176.1 cm with SD of 7.47 cm.

None of these standards is “better” than the other. The SAP standards are perhaps more useful for Argentine doctors to evaluate situations of food deprivation or growth retardation in a locality or in relation to an individual. The WHO 2007 standards indicate the gap between national or local heights and a growth potential determined by international height data. Though nations keep reporting stunting and waste indicators in relation to their national standards, for comparative purposes it is more useful to use the WHO standards. Because the WHO standards are higher than the national SAP standards, those high-for-age z-scores calculated using the former would become our upper-bound estimate of stunting, while those calculated with the latter would represent the lower-bound of stunting rates. After these calculations, we present a third estimate that uses as a standard the 75th centile of heights from the Golden Age period. Expectedly, this third standard produces results very close to our lower-bound estimates.

According to WHO [54] height-by-age measured in z-scores is the best indicator for chronic malnutrition (stunting) across the world. Only height can capture neatly the deficiencies in caloric intake and the insults of disease of early infancy. Children from quite different ethnicities and geographies, the World Health Organization claims, have in principle the same potential growth to achieve. In this study we apply the methodology suggested by the World Health Organization to measure chronic malnutrition in adult populations in the Argentine past. We define stunting (moderate and severe) as a situation in which height-by-age z-scores with regard to modern standards of growth are below minus two (HAZ < −2). We use two different standards of modern growth: one developed and approved by the Argentine Pediatric Society in 2001 [55]; the other is the WHO reference standards for adolescents’ heights published in 2007 [64].

Three of our five samples, (the sample taken from the 1927 Electoral Record, the Patricios Battalion sample; and the sample of army recruits born in 1916–1951) refer to 18-year-old recruits. To these samples, it is correct to apply the tables developed for evaluating the growth of adolescents, to the extent that these standards contain the normalized average height and standard deviation for males of that exact age (18 and 18.5). The other two samples refer to populations of ages 19 to 49 in the case of militiamen of the National Guard, and 21–54 in the case of inmates of Buenos Aires prisons. Is it reasonable to assume that these distributions can be compared with standards estimated for 18–19 year olds? The difference in stature between 19-year old militiamen and their comrades of 20–25 years of age proved not significant (the estimated coefficient was 0.35 cm), while difference in S.D. was negligible. So, even in the case of late 19th century men, where one could assume that growth continued into ages 19–20, the small difference in mean stature does not justify changing the standards of comparison. With regard to the data collected from Buenos Aires prison inmates, our sample have excluded prisoners younger than 21, removing in this way the bias that can result from including individuals who had not completed their adult height (true, the prison population from 15 to 19 presents a rather irregular height-by-age profile: a 15-year old is 6.4 cm shorter than a 20-year old; and 17-year old is only 1.5 cm shorter than a 20-year old. This could be the result of the police arresting 15-year-old petty thieves coming from the street-poor, while the prison population 20 and over belongs mostly to better-fed regular working-class. For prisoners of these particular institutions, studying adolescent catch-up would be quite risky. It is better to disregard these observations).

There are risks involved in estimating stunting rates based upon distributions of adult heights. These risks become manageable if we have a clear understanding about what the standards represent and what stunting rates are supposed to measure. If one sticks to the definition given by the World Health Organization, the estimated standard (whether it refers to children or adolescents) represents the health and nutrition status of children who had grown in the best possible socio-economic and family/health environment. In this regard, it may be applied to Ethiopian, Vietnamese as well as to French, Ukrainian, or Argentinian children. It is a universal standard. It does not represent the future genetic potential of human beings, for this information is not yet available. So, there are no grounds to argue that standards should be changing from one decade to another. For less developed or emerging economies, the standards are high enough to function as a growth potential for many decades. The concept of “stunting rate” is one of a relative deficiency (shortness) in relation to a higher universal standard. It was devised (together with other indicators) to measure the progress or stagnation of countries’ efforts to reduce malnutrition. If we keep changing the standards, we will never be able to measure net-nutritional progress.

Secondly, we need to understand that stunting is measured using the whole distribution of heights and not by looking only at the average level. The stunting rate is then an exercise of comparison between two distributions of heights, the actual and the standard. Over time, it is expected that both the mean and the SD will change, hence we need both indicators. The “stunting rate” is much like a poverty rate, it depends on the number of people (head count) that fall to the left of a benchmark or cut-off point (2 SDs from the mean or the 50th centile of the standard). Choosing a too high standard of potential growth will result in an overestimation of stunting, the same way as choosing the wrong poverty-benchmark can produce quite erroneous results about poverty. The famous “two dollar a day” benchmark can dramatically underestimate poverty rates (in many countries it takes probably five times that amount not to fall into extreme poverty). Argentine doctors, members of the S.A.P. committed for re-estimating height standards, believed that WHO 2006 standards overestimated malnutrition among Argentine children.

Finally, let me address briefly the question of catch-up growth. One might reasonably assume that the distribution of heights at age 20 is a simple transformation of the distribution of heights of that same population measured at age 13. Here, the main altering factor would be catch-up growth. Stunting rates use two indicators to sum up the information of a distribution: the mean and the standard deviation. If there is adolescent catch-up, would this change the mean and standard deviation? Would this be a substantial change? In my opinion the available evidence is that adolescent catch-up affects so small proportion of a population that it is unlikely to change significantly the distribution of adult heights. If there is catch-up growth the stunting rate measured at age 18 or 20 should be smaller than that measured at 13 or 10. Yet in some cases, the contrary has occurred. Wang, Popkin and Zhai report that among Chinese adolescents the stunting rate of 14–18 year-olds was 7.1 percentage points higher than that measured on 10–13 year-olds. [65].

The evidence about catch-up growth if far from conclusive (though there are antecedents, many studies were stimulated by the classic essay by Prader, Tanner and von Harnack [66]). In a survey of the literature made in the early 1990s, Golden et.al. found that there was evidence of catch-up growth among stunted children who had been adopted, emigrated, or treated for debilitating disease. Yet this catch-up was partial. Stunted children may reach their individual growth potential (given by parental height), but not converge towards the height of affluent societies—the latter achievement, the authors said, would require generations. [67] Adair found that among Filipino children who were stunted at age 2 thirty percent recovered from stunting at age 8.5, and 32% were no longer stunted at age 12. In the best of cases, catch-up was a selective process. Children with taller mothers, those who were longer at birth, and those who were less severely stunted had better chances for recovering [68]. A 14-year longitudinal study conducted among Australian children showed that very-low-birth children had a capacity for catch-up from ages 2 to 14, but only to the growth potential of their parents. At age 14 children born with very low birth weight (w < 1000 gr) were still significantly shorter than children with normal birth weight. (At age 2 VLBW children were 3.4 cm below NBW children, and at age 14 the difference had increased to 6.1 cm) [69]. A recent study by Himaz has complicated the picture arguing that children in India between the ages of 8 and 19 may move in or out of stunting. Some children of Utah Pradesh seem to have attained partial catch-up growth, yet there were other children who moved in the opposite direction. Half of stunted children at 19 had acquired that condition during mid-childhood and adolescence. Looking only to those who were stunted at 8 and following them to age 19, it would appear that the stunting rate declined from 30% to 14%, but in actuality, the newly added layers of stunted children and adolescents raised the final stunting rate to 25% (the net decline was only 5 percentage points) [70].

The “considerable fluidity” in children’s stunting profiles makes it difficult to extrapolate to other societies the net impact of childhood stunting upon young adult stunting. If there is adolescent catch-up grow (which I am sure there is), there are reasons to argue that the stunting estimated from adult heights underestimates the rates of stunting at ages 5, 8, or 12. But, on the other hand, there are also good reasons to believe that this underestimation would be low. Particularly in a country or region rich in food production, unaffected by massive poverty, and with declining infant mortality rates—as the Argentine Pampa region was—one may expect that the extent of catch-up growth in terms of percentage of the population will be small. Alone the same lines, one may expect that sustained improvements in sanitary conditions and public health would reduce stunting rates substantially, altering the distribution of adult heights in proportion. Unfortunately, present-day Argentina does no longer correspond with this description (Argentina is now a country with a stagnant economy, with substantial poverty and unemployment, and with increasing child malnutrition) and, consequently, the question for the second half of the 20th century would be: when and where did stunting rates started to grow again? But this is a completely different story, one that needs to be addressed in a separate study.

## 5. Main Findings

**Figure 1:**
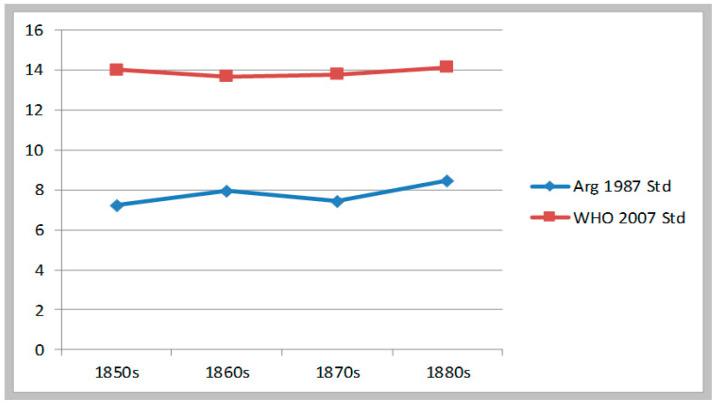
Evolution of moderate & severe stunting. National Guards b1855–1883 (in%).

Following this methodology, we estimated moderate and severe malnutrition rates for each sample. Table 2 presents the estimates per decade for the National Guards (1857–1883). For the whole period, stunting stood at 7.7% according with the 1987 Argentine standard, and 13.8% according to the WHO 2007 standard of growth. Using the Argentine benchmarks, the stunting rate rose 1.2 percentage points between the 1850s (7.24%) and the 1880s (8.45%), while using the WHO benchmarks the stunting seemed to have remained stable at the 14% level. As shown by this evidence, in this early period in spite of a great expansion in land appropriation, farming, and cattle- and sheep-raising, conditions of health and nutrition in Buenos Aires province were not improving; or perhaps, even showing a mild deterioration. See Figure 1. 

**Figure 2:**
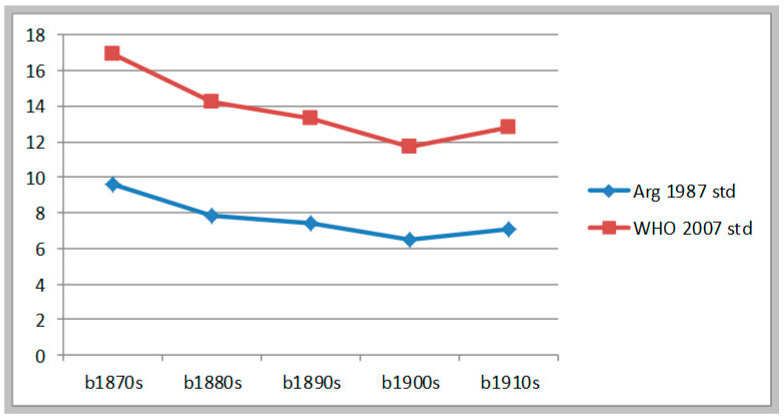
Evolution of moderate & severe stunting. Citizen-soldiers from the Pampa Region b.1874-b.1910.

The sample of citizen-soldiers taken from the Electoral Registration of 1927 brings a different picture of the following period. Though the rates of stunting are similar to the previous sample (7.5% under Argentine standards and 13.5% under WHO standards), here we find a clear declining tendency throughout most of the period. Using the Argentine 1987 standards the stunting rate declined 3.1 percentage points from the 1870s to the 1900s (from 9.57% to 6.47%), and then increased from the 1900s to the 1910s, generating a net gain of 2.5 percentage points for the whole period. (See Table 3). If we instead use the WHO 2007 standards, the stunting rate declined 5.2 percentage points from the 1870s to the 1900s and then went up in the following decade (from 16.89% to 11.72%) to finally increase to 12.79% in the 1910s. Thus, the net gain for the period 1870–1919 was 4.1 percentage points. This sample represents the biological evolution of male inhabitants of the Pampa region. For them, health and nutrition conditions improved significantly from 1870 to the 1909, yet there was a minor setback in the 1910s (this finding is consistent with what I have called a situation of “nutrition stress” in the period 1907–1912 [5]). See Figure 2.

**Figure 3:**
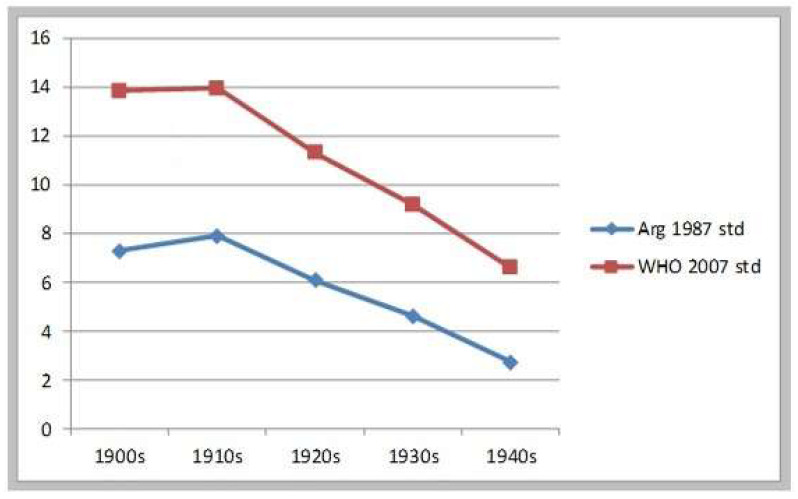
Evolution of moderate & severe stunting. Recruits from Regiment Patricios b1901–1943.

In short, health and nutrition conditions for children did not deteriorate—as it is generally assumed—during the great agrarian expansion of 1870 to 1914. On the contrary, malnutrition decline during most of this period. The following sample represents the health and nutrition conditions of recruits residing in the neighborhood of Buenos Aires city, most of them born in the littoral and the interior provinces (between 1901 and 1943). This sample shows a much improved situation towards the 1930s and 1940s. Using the Argentine 1987 standards, the malnutrition rate shows a small increase from the 1900s to the 1910s, and from then a remarkable reduction of 4.6 percentage points until the early 1940s. (See Table 4) Measured against the WHO 2007 benchmark, the reduction in malnutrition was even more impressive: 7.4 percentage points between the 1910s and the early 1940s—remaining basically stable between the 1900s and the 1910s. Over the whole period 1901–1943 the malnutrition rate was reduced to a third or to half of its value, according to the standard we use. Under both estimates then, the improvement in conditions of early childhood after 1910 was quite impressive. See Figure 3.

**Figure 4:**
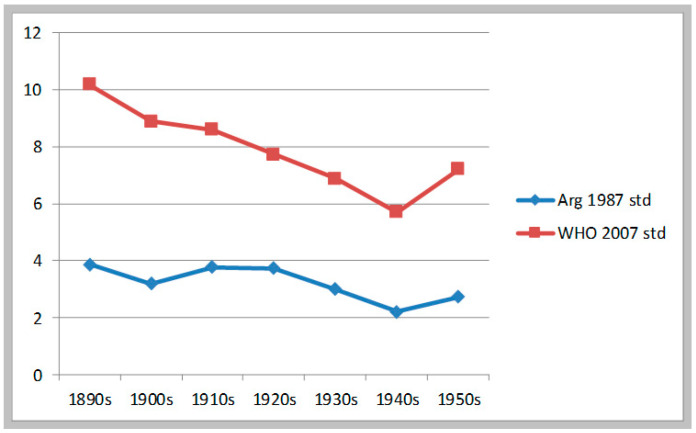
Evolution of moderate & severe stunting. Inmates of Buenos Aires Prisons, b.1885–1959.

Estimates of malnutrition derived from prisoners’ heights confirmed the previous findings. Since this is the longest of our series, we can see here most of the picture. Estimates based on WHO standards suggest that the decline in malnutrition was sustained from the 1890s to the 1940s: the reduction was 4.5 percentage points (from 10.17% to 5.7%)—there was no rebound in the 1910s as in the previous sample. After that, malnutrition increased from the 1940s to the 1950s (from 5.7% to 7.21%). (See Table 5) Estimates based on Argentine standards tell a somewhat different story: they show deterioration from the 1900s to the 1910s and then stagnation into the 1920s, and only then a decline from 1920 to 1939. In these estimates, the number of cases below the −2 SD mark are very low, so any skewedness in the distribution may produce errors (curiously, the prisoners’ data show that imprisoned delinquents, though solidly working-class in origin, were better-fed and healthier than army recruits). See Figure 4.

**Figure 5:**
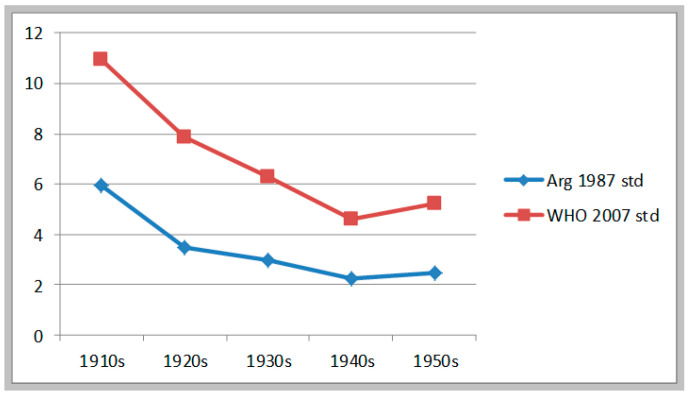
Evolution of moderate & severe stunting. Recruits from Pampa Region b.1916–1951.

Finally, Table 6 and Figure 5 show the estimates for malnutrition rates that result from the sample from army recruits of the Pampa region, between birth cohorts 1916 and 1951. Estimates derived using the two standards produce different stunting rates: 3.5% on average, using Argentine standards; and 7.1% on average, using WHO standards. Yet the trend is similar. These estimates indicate a continuous decline in malnutrition rates from the 1916 cohort to the 1943 cohort, a reduction of 3.7 percentage points using Argentine benchmarks, and of 6.4 percentage points using WHO benchmarks. And from 1943 to 1951 they show a minor increase in malnutrition rates (less than 1 percentage point in both cases). The long interwar period, these estimates are saying, was for the residents of towns and cities of Buenos Aires province a time of sustained improvement in health and nutrition among children. The period after 1943 (a transition military regime first, and then the first Peronist administration) was not so good: malnutrition either stagnated or mildly increased (we have found a similar phenomenon using estimates of stature in the Buenos Aires Conurbano (the capital´s surrounding industrial cities). (Salvatore 2009)

**Figure 6:**
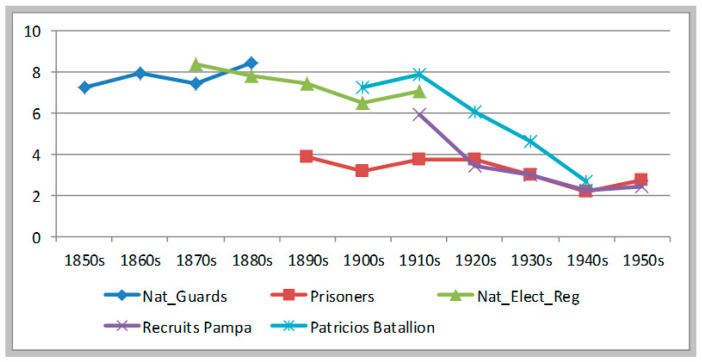
Evolution of malnutrition rates in Argentina (1850s to 1950s). Calculated based on Argentine 1987 standards.

**Figure 7:**
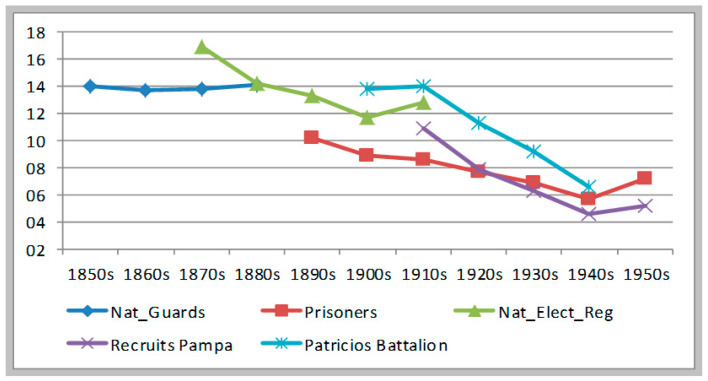
Evolution of malnutrition rates in Argentina (1850s to 1950s). Calculated based on WHO 2007 standards.

**Figure 8:**
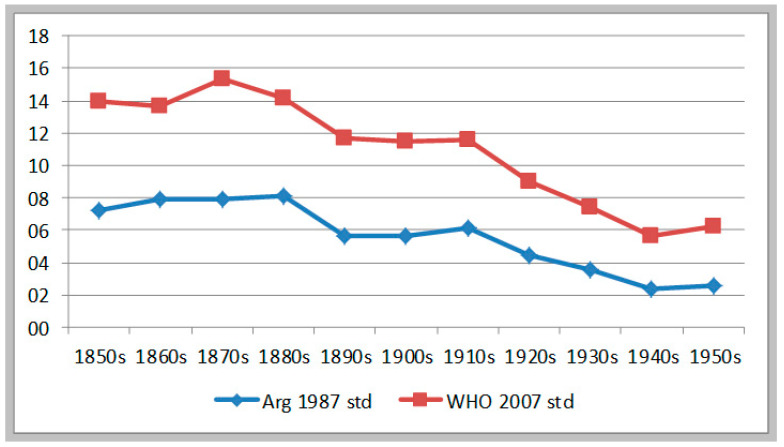
Moderate and severe stunting in the Pampa Region.

Figure 6, Figure 7 and Figure 8 summarize our findings. The first two figures simply present together our five series, according to the two standards of modern growth (Argentine 1987 and WHO 2007). Figure 8 shows the simple average of these two estimates. Clearly, the Argentine 1987 standard produces a lower estimate of malnutrition (HAZ < −2) than the WHO standard. The former should be taken as a lower-bound and the latter as the upper bound of the true stunting rate. I am inclined to think that the true stunted rate would be closer to estimates using the WHO standards—that is closer to our upper-bound, rather than to our lower-bound estimates. Why? Because the values attained using the Argentine 1987 benchmark are too low for the 1940s and 1950s (2.4% and 2.5%), whereas the malnutrition rate using the WHO standard ranges from a maximum of 15.3% (1870s) to a minimum of 5.6% (1940s). From the 1850s to the 1880s the stunting rate was either stable or moderately rising (if so, there is ambiguity about whether the peak of malnutrition was reached in the 1870s or in the 1880s). From the 1880s all the way into the 1950s, both estimates show the same declining trend, except for a period of rebound between the 1900s and the 1910s.

Based on the best available distributions of stature referring to male recruits, militiamen, prisoners, and citizen-soldiers of Buenos Aires province and the Pampa region, we can affirm that stunting rates increased moderately between the 1850s and the 1870s and that afterwards, they declined in a sustained way (perhaps with some rebound in the 1910s) into the 1940s and 1950s. The initial increase in malnutrition (1.3 percentage points) was minor compared with the large reduction of malnutrition in the period 1870s to 1950s. Taking the estimates generated using the WHO standards, the stunting rate declined 9.1 percentage points between the 1870s and the 1950s (from 15.3% to 6.2%); a remarkable achievement for the period and the region.

## 6. Are These Estimates Reasonable? How Precise Are They?

Robustness is perhaps too a strong a term. With historical evidence, sometimes one can be very precise; more often on cannot. Hence, we wanted to prove the consistency of our findings by refining some aspects of our estimations. First, we divided into two groups the populations under scrutiny: on one side the recruits and citizen-soldiers who were about 18–19 years of age at examination; on the other hand, the militiamen and the prisoners who were older than 20. With the first group (18–19 year-olds) we limited the sample to include only populations 18.0 to 18.9 years of age. With the second group, we retained only observations of people ages 20 to 45. In this way, our sample populations became more restricted and more homogenous—hence, more comparable.

In addition, we separated our information into three periods, to correspond the first to what Argentine historians call the period of “national organization” (1850–1885), the second to the Golden Age, otherwise called the era of export-led growth (1885–1914), and the third to a period characterized by rapid urbanization, industrialization, and increasing social and income inequalities (1915–1959). Argentina is a country where the best period in terms of wealth and wellbeing is in the early twentieth century, not in the 1950s. Hence, it is in the second period that we see the start of significant improvements in health, as a result of better urban sanitation and the expansion of medical care, especially for mothers and children. And this coincided with a significant expansion of food availability.

**Figure 9:**
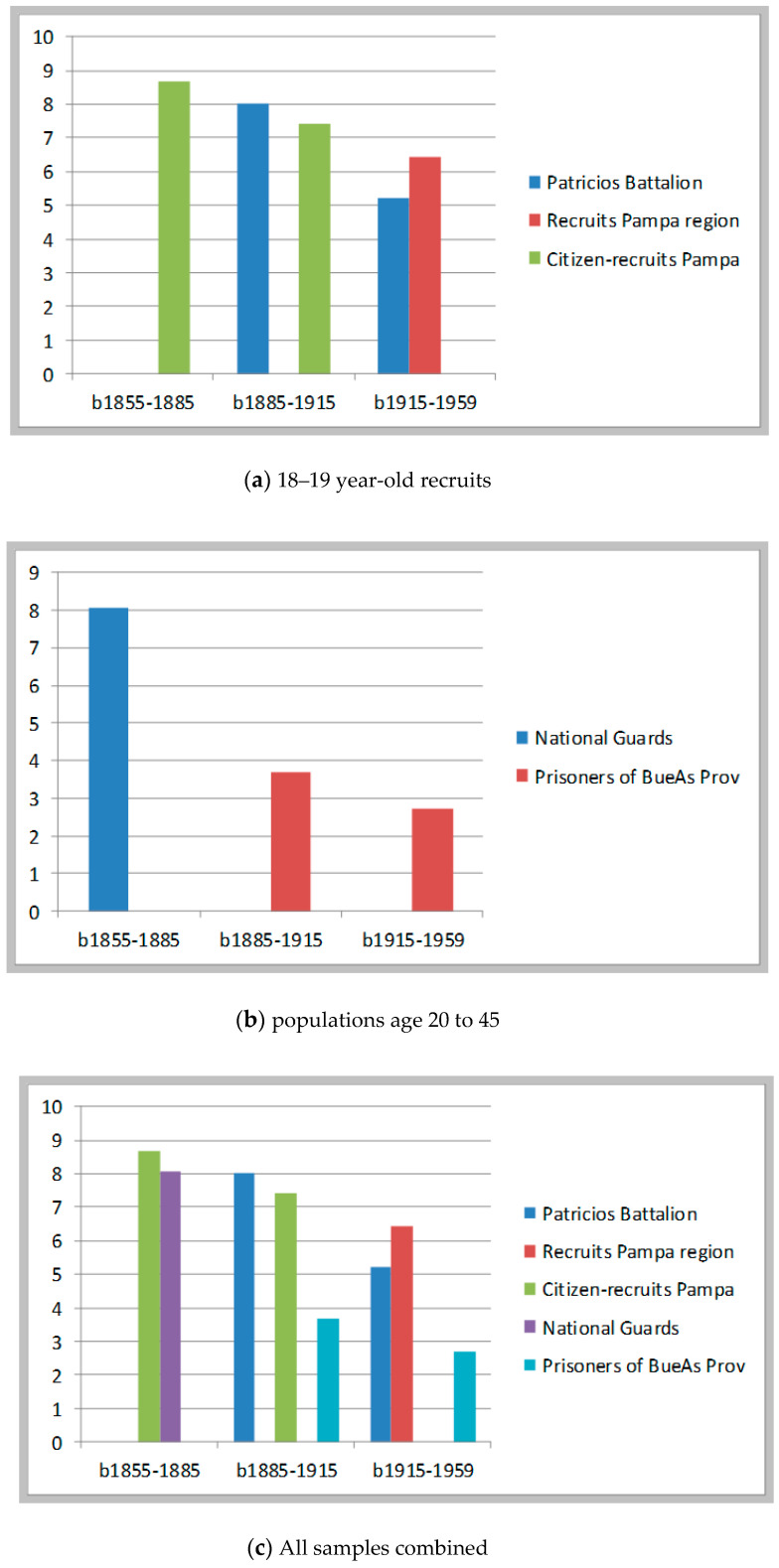
Estimated stunting rate—using 75th height centile as standard.

Finally we re-estimated stunting rates using as a benchmark, the 75th centile corresponding to the age of progress (1885–1815); that is, 172.5 cm with a SD of 6.282. The results of the exercise are summarized in Table 7 and Figure 9.

One interesting finding is that using a fixed benchmark, the biological wellbeing of the poor and stunted is remarkably the same over time: 156.2 to 156.8 cm. This is very revealing finding. It means that whatever were the actual gains in biological wellbeing in this near 100 years, the children affected by stunted had the same stature in adulthood; in the 1850s as in the 1950s. The rate of stunting over the long-run, however, tended to decline, indicating an improvement of health and nutrition among the general population—not among the stunted. This finding is consistent with the idea that in a country with high and increasing social and economic inequalities, such as Argentina, there is a persistent or structural poverty—and if you will, a “poverty biological trap”—that keeps some part of the population equally poor and malnourished.

These estimated rates of stunting are low, very close to those calculated using national SAP standards and about 4–5 percentage point lower than those calculated with WHO 2007 standards. This was to be expected. As it turned out, the 75th height centile of the period 1885–1915 is quite similar to the SAP standard (this itself is remarkable: the 75th centile of heights in the era of export-led growth was still considered the appropriate benchmark to evaluate children’s growth by Argentine pediatricians in the 1990s). The overall trend is, however, similar. These estimates—like those presented in Figure 8—show a long-term decline of stunting rates over the period 1855–1885 to the period 1915–1959. Differences in stunting rates calculated using different samples are quite small, ranging from 0.6 to 1.2 percentage points. I would say these populations produce quite similar stunting rates, despite some minor differences in sample composition. The only population that is truly peculiar is the sample of prison inmates from Buenos Aires: they are taller than average and show significantly lower stunting rates. Hence, if I were to put my doubts in the comparability of the samples this would be the one.

In addition, we conducted the experiment of estimating stunting rates with changing standards over time (that is, using the 7th centile height of each sample). The 75th centile increased from 172.0 cm in the 1870s to 175.0 cm in the 1950s, more than the increase in Argentine average heights. This experiment led to inconsistent results: the 18–19 year-old populations showed similar long-term decline in stunting rates, but the populations 20 to 45 showed stunting rates that were either stagnant or increased over time. Since there is no valid reason for these anomalous results, we concluded that they were the artifact of using movable standards. Hence, we went back to the original method of using the same standard over time. In addition, we tried adding one centimeter to the 75th centile standard to estimate the stunting rates of populations ages 25 to 40. The declining trend was maintained while the stunting rates went up about 1 to 2 percentage points. In addition, the mean height of all stunted populations went up, about 1.7 cm in average (to 158.1 cm).

## 7. Brief Discussion of Findings

While there is much to refine about these estimates, this important long-run decline in malnutrition rates in Buenos Aires and the Pampa region (1870–1959) needs further reflection and analysis. Was this a consequence of rising food availability at reduced real cost? Was this the result of government sanitary and health policies? Was this the product of an effective reduction in child labor? Or was this reduction in stunting rates simply the overall effect of increasing real family incomes? To answer these questions will demand sustained and well-focused research on these topics (food availability, food prices, health policies, infant mortality rates, child labor, and real family income) and this was not the object of this paper. This paper was intended to estimate long-run stunting rates. By producing estimates based on two different standards of modern growth, I have provided researchers with a lower and an upper bound estimate of this rate. According to the upper bound (estimates based on WHO 2007 standards), perhaps the more reliable, malnutrition declined from 14.0% in the 1850s to 6.2% in the 1950s, for male individuals raised in the Pampa region or in Buenos Aires province.

Health and nutrition are probably the chief underlying factors behind this long-term decline in stunting rates, although there was also a decline in child labor to be considered. For countryside and towns of Buenos Aires province, there are no long-term estimates of infant mortality. They are only available for the capital city. According to the evidence provided by Mazzeo, the city’s infant mortality rate (0–4) declined from 350 per thousand born alive in 1860–1864 to 172 per thousand in 1900–1904; and continued falling in the 20th century. The IMR (0–4) was 91.7 in 1930–1934 and 39.8 in 1955–1959 [68,71]. We do not know how rapidly this improvement in children’s health in the Capital was extended to the province of Buenos Aires and the other areas of the Pampa region. We have solid estimates of IMR (0–1) for the provinces of the Pampa region for the more limited period of 1914–1953. Between these two years the IMR (0–1) for the province of Buenos Aires declined from 92.2 to 53.5. The other provinces of the region have important declines as well: Santa Fe went from 142.2 to 49.4 per thousand; Entre Rios moved from 112.2 to 63.9; and Córdoba from 152.1 to 71.2 per thousand. During this period (1914–1953) the fall in the IMR at the Capital city was spectacular: it dropped from 94.3 per thousand to 35.5 per thousand [15,16,17,18,19,72].

These important improvements in children’s health are difficult to imagine without the increased availability of food (a result of the Great Agrarian Expansion of 1880–1920). This was probably the basis of the sustained decline in stunting rates. Apparently, the export bonanza of 1875–1914—assisted by the expansion of farming and livestock, the significant growth of the railroad network, and the massive inflow of European immigrants and capital—had a beneficial effect on the biological wellbeing of the region’s children. The stunting rate declined during most of this period, probably the result of a combination of a greater availability and variety of food and the healthy conditions of small towns and cities of the Pampa region. Farmers in the Pampa region generated abundant harvests of corn and wheat and provided urban markets with sufficient beef and mutton—yet at the cost of increased use of child labor. Though the city of Buenos Aires began to improve its sanitary conditions and health service, particularly after the yellow-fever epidemic of 1871, progress in this area was probably modest before 1910. And it is likely that the extension of sanitary reform took time to reach the towns of the Buenos Aires countryside and the other provinces of the region. Between 1905 and 1915 there was stop and reversal in the decline in malnutrition, probably due to increasing food prices, greater competition in labor markets, and the intensified use of child labor. In an earlier article, I have shown the existence of a situation of “nutrition stress” due to the oversupply of immigrant labor and the fall of real wages [5].

The First World War was a period of shortages of imported foodstuffs, inflation, and declining real wages. Hence, the reduction of malnutrition must have occurred after 1918. Starting in the 1920s, there was an impressive and sustained decline in malnutrition rates due to multiple factors. Among them we could mention; early industrialization and its impact on job creation; the decline of food prices in the late 1920s and the 1930s; and a series of regulations aimed at reducing child labor and improving urban sanitation. The provision of food to school children started in the city of Buenos Aires in the early 1930s, and probably extended to other towns and cities in the 1940s and 1950s. The small rise in the malnutrition rate in the 1950s gives us a warning about the limitations to the redistributive policies of Peronist administration (1946–1955) in comparison to the wellbeing attained in the 1930s and early 1940s (only the prison data suggests that the reduction of stunting lasted five decades, yet the distribution of this sample is less reliable in its left-hand side than comparable samples of army recruits and militiamen).

With regard to the traditional periodization of Argentine economic history, these estimated rates of malnutrition provide also a new viewpoint. Both types of growth (export-led growth and import-substituting growth) were beneficial to biological wellbeing: they tended to reduce the stunting rate in Buenos Aires and other provinces of the Pampa region. This favorable effect lasted two or three decades (1870–1899 or 1880–1909) during export-led growth, according to whether we consider the five-sample average or the sample or citizen-soldiers from Pampa region. The beneficial effect of industrialization in this region lasted instead four decades (from 1910 to the 1949), no matter which combination of samples we use. As I have argued elsewhere [16], industrialization and rapid urbanization during the interwar period brought about increasing inequalities in net-nutrition.

## 8. Conclusions

Throughout the period 1850–1950, native Argentines raised in Buenos Aires province or in the Pampa region were relatively well-fed, in relation to both national and international standards of growth. In the earlier period (1850–1910) the rates of moderate and severe malnutrition (HAZ < −2) remained above 10% (ranging from 14.0% in the 1850s to 11.62.2% in the 1910s), levels that the World Health Organization (WHO 2018) considers “medium” malnutrition” in global comparative standards. From the 1920s to the 1950s the estimated rate of malnutrition (considering only the upper-bound estimate) remained below 10% and greater than 2.5% (declining from 8.9% in the 1920s to 6.2% in the 1950s), levels that the same organization considers “low malnutrition”. If we instead contrast our estimates against the less-demanding classification recommended by WHO in its well-known 1995 technical paper (“Physical Status”), all of our stunting rates would be considered “low” (under 20%), including those estimates for the 1850–1870 cohorts.

Our estimates are indicators of a well-fed population; that is, a situation in which the majority of children had access to sufficient caloric intake and relatively good health; while only a minority (less than 10 percent after 1920) showed signs of chronic malnutrition. In fact, one could argue that already in the 1940s and 1950s the Pampa region was getting close to the status enjoyed by the more developed economies (“very low malnutrition,” for countries with less than 2.5% stunted children). How close? 2.5 to 3 percentage points. Hence, we are ready to solve our first puzzle: the native population to the Pampa region was subject to low or medium malnutrition, regardless of whether we are referring to the period of export-led development (1880–1920) or to the era of state-led industrialization (1930–1960). Again, this finding does not refer to the interior of Argentina or to the poor surrounding industrial towns of Buenos Aires city (for information on the evolution of biological wellbeing in the Northwest, see [17]).

The second conclusion relates to the evolution of malnutrition over time. Due to multiple factors, Argentina’s rate of malnutrition declined from 15.3 in the 1870s to 5.6% in the 1940s, even when using the upper-bound estimate. That is, malnutrition was reduced to almost a third in the course of eight decades. This seems a remarkable achievement, for a nation that had experienced rapid and dramatic transformations in its economy, society and politics. At first (1850–1880), the opening to world markets seem to have worsened the condition of malnutrition, yet soon the trend was reversed and the export-boom of the period 1880–1914 brought about increasing real income and wages, relatively stable food prices, and an improvement in the disease environment. After a short period of stagnation in average heights and malnutrition in the early 1910s, the country returned to a similar dynamic of declining malnutrition, this time under new social, political and economic conditions. 

The dramatic fall of European immigration during WWI was followed by a remarkable export performance in the 1920s that raised incomes and employment. The closing of world markets for Argentine grain and beef during the Great Depression brought about declining food prices for domestic consumers, whereas industrialization in the Great Buenos Aires area provided employment for rural-urban migrants. The conservative governments of the 1930s continued the work of their predecessors, extending sanitation improvements (sewage works and drinking water) and medical care (public hospitals) to more cities. Thus, the double process of industrialization and rapid urbanization, instead of generating a situation of impoverished quality of life—as it had been the case in the industrialization of the United States and Northern Europe in the 19th century—produced an improvement in the biological standard of living and a consequent sustained decline in malnutrition rates. As infant mortality rates suggest, the improvement of the health environment might have facilitated this sustained reduction in stunting rates. And so did the Great Agrarian Expansion of 1880 to 1920 which significantly increased the per-capita availability of food. Yet more research is required to assess the intricate linkages between rising income and agricultural productivity, declining infant mortality, the control of child labor, and the decline in malnutrition among children and adolescents. 

## Figures and Tables

**Table 1:** Five samples: basic statistics.

	Sample	No. Observ.	Mean Stature	Std. Dev.	Min.	Max.
1	National Guards b.1857–1883	11,158	168.0	6.158	137	195
2	Citizen-soldiers Pampa b.1874–1910	20,925	168.21	6.274	141	196
3	Recruits. Patricios Battal. b.1901–1943	29,430	169.1	6.364	140	197
4	Inmates. Bue As Prisons b.1885–1959	12,873	169.88	6.083	150	194
5	Recruits. Pampa Reg. b.1916–1951	10,112	170.65	6.427	165	196

Sources: see text.

**Table 2:** Stunting rates by decade National Guards b.1857–b.1883.

Decade of Birth	Stunting Rate Argentine 1987 Standard HAZ < −2 Cases	Percentage	N =	Stunting Rate WHO 2007 Standard HAZ < −2 Cases	Percentage
1850s	74	7.24	1022	143	14.0
1860s	421	7.95	5298	725	13.68
1870s	319	7.43	4293	592	13.79
1880s	46	8.45	544	77	14.15
Total Sample	860	7.71	11,128	1537	13.77
N = 11.128					
Argentine 1987 standards		Mean = 172.7 cm		SD = 6.83 cm	
WHO 2007 standards		Mean = 176.1 cm		SD = 7.47 cm	

**Table 3:** Stunting rates by decade Citizen-soldiers from Pampa Region b.1874–b.1910.

Decade of Birth	Stunting Rate Argentine 1987 Standard HAZ < −2 Cases	Percentage	N =	Stunting Rate WHO 2007 Standard HAZ < −2 Cases	Percentage
b1870s	258	9.57	2694	455	16.89
b1880s	396	7.86	5038	716	14.21
b1890s	465	7.43	6259	832	13.29
b1900s	375	6.47	5793	679	11.72
b1910s	81	7.1	1141	146	12.79
period b1874–1910	1575	7.53	20,925	2828	13.51
N = 20,925					
Argentine 1987 standards		Mean = 172.7 cm		SD = 6.83 cm	
WHO 2007 standards		Mean = 176.1 cm		SD = 7.47 cm	

**Table 4:** Stunting rates by decade Patricios Battalion b.1901–1943.

Decade of Birth	Stunting Rate Argentine 1987 Standard HAZ < −2 Cases	Percentage	N =	Stunting Rate WHO 2007 Standard HAZ < −2 Cases	Percentage
1900s	313	7.29	4294	595	13.86
1910s	497	7.91	6286	878	13.97
1920s	488	6.09	8007	907	11.33
1930s	353	4.62	7642	702	9.18
1940s	87	2.72	3201	211	6.59
b.1901–1943	1738	5.9	29,430	3293	11.19
N = 29.430					
Argentine 1987 standards		Mean = 172.7 cm		SD = 6.83 cm	
WHO 2007 standards		Mean = 176.1 cm		SD = 7.47 cm	

**Table 5:** Stunting rates by decade Inmates of Buenos Aires prisons b.1885–1959.

Decade of Birth	Stunting Rate Argentine 1987 Standard HAZ < −2 Cases	Percentage	N =	Stunting Rate WHO 2007 Standard HAZ < −2 Cases	Percentage
1890s	27	3.87	698	71	10.17
1900s	57	3.20	1778	158	8.89
1910s	113	3.77	3001	258	8.60
1920s	93	3.74	2485	192	7.73
1930s	28	3.00	932	64	6.87
1940s	40	2.21	1806	103	5.70
1950s	57	2.74	2078	150	7.21
Total Sample	418	3.25	12,778	1005	7.81
N = 12.778					
Argentine 1987 standards		Mean = 172.7 cm		SD = 6.83 cm	
WHO 2007 standards		Mean = 176.1 cm		SD = 7.47 cm	

**Table 6:** Stunting rates by decade Recruits from Pampa Region b.1916–1951.

Decade of Birth	Stunting Rate Argentine 1987 Standard HAZ < −2 Cases	Percentage	N =	Stunting Rate WHO 2007 Standard HAZ < −2 Cases	Percentage
b.1916 (1910s)	133	5.93	2244	246	10.96
b.1929 (1920s)	70	3.46	2020	159	7.87
b.1934 (1930s)	56	2.97	1887	119	6.31
b.1943 (1940s)	44	2.23	1973	91	4.61
b.1951 (1950s)	49	2.46	1988	104	5.23
period 1916–1951	352	3.48	10,112	722	7.14
N = 10,112					
Argentine 1987 standards		Mean = 172.7 cm		SD = 6.83 cm	
WHO 2007 standards		Mean = 176.1 cm		SD = 7.47 cm	

**Table 7:** Stunting rates estimated using the 75th centile as standard.

Period and Sample	Benchmark	Stunting		Mean	Mean Height
	75th Centile	Head Count	Rate (%)	HAZ	Stunted Pop
**(1) Populations Age 20–45**					
National Guards					
b.1855–1883	172.5	887	8.06	−2.499	156.79
Prisoners of BueAs Prov					
b.1885–1915	172.5	130	3.67	−2.541	156.34
b.1915–1959	172.5	187	2.7	−2.537	156.56
**(2) Populations Age 18–19**					
Patricios Battalion					
b.1901–1915	172.5	625	8.02	−2.593	156.21
b.1915–1943	172.5	1161	5.22	−2.549	156.48
Recruits Pampa region					
b.1916–1951	172.5	336	6.42	−2.594	156.2
Citizen-Recruits Pampa					
b.1874–1885	172.5	548	8.67	−2.552	156.46
b.1885–1910	172.5	1120	7.41	−2.552	156.46

*Notes*: Benchmarks for all samples = 172.5 cm; SD = 6.282 cm. Source: Own estimates based on the five samples.
